# Digital biomarkers as predictors of brain injury in neonatal encephalopathy

**DOI:** 10.3389/fped.2025.1617155

**Published:** 2025-08-29

**Authors:** Nahla Zaghloul, Neel Kamal Singh, Weihuang Xu, Kaitlyn Lagnese, Livia Sura, Juan Carlos Roig, Mehmet Albayram, Dhanashree Rajderkar, James L. Wynn, Alina Zare, Michael D. Weiss

**Affiliations:** ^1^ Division of Neonatology, Department of Pediatrics, University of Florida, Gainesville, FL, United States; ^2^ Department of Electrical and Computer Engineering, University of Florida, Gainesville, FL, United States; ^3^ Department of Radiology, University of Florida, Gainesville, FL, United States

**Keywords:** neonatal encephalopathy, therapeutic hypothermia, digital biomarkers, brain injury, long short-term memory (LSTM) neural network, machine learning models

## Abstract

**Background:**

Neonatal encephalopathy (NE) is a significant cause of neurodevelopmental impairment, with therapeutic hypothermia (TH) being the current standard of care for mitigating brain injury in affected neonates. Despite advances, there is a critical need for early, reliable biomarkers that can predict brain injury severity and long-term outcomes, particularly during the 72-h hypothermia window. This study explores the potential of digital biomarkers derived from continuous bedside physiologic monitoring to predict MRI-confirmed brain injury in neonates with NE.

**Methods:**

We collected continuous physiologic data from 138 neonates undergoing TH, including heart rate, systemic oxygen saturation (SpO₂), cerebral oxygen saturation (rcSO₂), systolic and diastolic blood pressure, and mean arterial pressure (MAP). Using a Long Short-Term Memory (LSTM) neural network, we developed predictive models to classify neonates into no/mild or moderate/severe brain injury groups based on MRI findings. Model performance was evaluated at 24 and 48 h of data collection. An ablation study was conducted to assess the relative importance of individual biomarkers.

**Results:**

Seventy-three neonates (52.9%) were classified as having moderate/severe injury, while 65 neonates (47.1%) had no/mild injury on MRI. The predictive accuracy of the LSTM model improved significantly with extended data duration, achieving an accuracy of 91.2% at 48 h compared to 84.6% at 24 h. The ablation study identified heart rate as the most significant biomarker, whereas rcSO₂ trends showed potential but did not consistently contribute to prediction accuracy in later models.

**Conclusion:**

Our study highlights the potential of digital biomarkers in predicting brain injury severity during the therapeutic hypothermia window. Machine learning models, such as LSTM networks, offer an opportunity for real-time prediction and risk stratification, ultimately enhancing clinical decision-making and neuroprotective strategies in neonates with NE. Future studies will focus on integrating real-time data capture and improving predictive accuracy.

## 1 Introduction

The incidence of neonatal encephalopathy (NE) is 1.5% per live birth in developed countries and varies between 2.3% and 26.5% in developing regions (1). NE is a major contributor to neurodevelopmental impairment (NDI) in children (2). The standard management for NE includes systemic supportive care and therapeutic hypothermia (TH), with total body hypothermia (TBH) shown to benefit approximately 20% of neonates with moderate to severe NE (3).

MRI with diffusion-weighted imaging (DWI) is the ideal diagnostic modality for early detection of brain injury in neonates with NE, with optimal imaging performed 4–5 days after birth, immediately following rewarming (4). However, logistical challenges often prevent MRI during the 72-h cooling phase, prompting the need for alternative bedside biomarkers that can predict both the extent of brain injury and long-term outcomes at 18–24 months of age.

Biomarkers are defined by the FDA-Joint Council as a “Defined characteristic that is measured as an indicator of normal biological processes, pathogenic processes, or biological responses to an exposure or intervention, including therapeutic interventions. Biomarkers may include molecular, histologic, radiographic, or physiologic characteristics” (5, 6). Potential sources of biomarkers include biofluids (e.g., serum, plasma, urine, cerebrospinal fluid), neuroimaging modalities like magnetic resonance spectroscopy (MRS), and digital biomarkers derived from physiologic monitoring such as amplitude-integrated EEG (aEEG), vital signs, and cerebral oximetry (7). Although biofluid biomarkers—such as neuroproteins, microRNAs (miRNAs), exosomes, and inflammatory cytokines—have received considerable attention, digital biomarkers remain relatively underexplored in predicting neonatal brain injury during hypothermia therapy (8, 9).

Advanced machine learning techniques, including random forests and deep learning, have been utilized to analyze EEG data for early seizure detection and to assess the severity of NE (10). When integrated with MRI-derived radiomic features, these approaches can accurately predict neurodevelopmental outcomes at 18 months, offering valuable insights for prognosis and guiding potential interventions (11).To date, most published trials on the use of machine learning (ML) in neonatal encephalopathy (NE) have not adequately addressed key structural issues related to design, data processing, applicable models, and validation and evaluation standards (12). Mooney et al. utilized the random forest machine learning algorithm and five-fold cross-validation to predict NE in a prospective cohort of infants with perinatal asphyxia, using maternal and delivery details along with the infant's condition at birth (13). Tian et al. developed and validated an intelligent NE identification model, called the deep learning clinical-radiomics nomogram (DLCRN), based on conventional structural MRI and clinical characteristics (14). Their study concluded that their model could expedite early mild HIE screening, improve the consistency of NE diagnosis, and guide timely clinical management. Lew et al. created a deep learning algorithm to predict 2-year neurodevelopmental outcomes in neonates with NE using MRI and basic clinical data such as sex and gestational age at birth (15). Their model focused on employing deep learning analysis of neonatal brain MRI to predict 2-year neurodevelopmental outcomes. None of the aforementioned studies or others were designed to utilize hemodynamic data recorded within the first 24–48 h and link these data to neonatal outcomes using an AI approach.

Given the large volume of data generated by bedside physiologic monitoring, artificial intelligence (AI) has emerged as a powerful tool in advancing clinical care by enabling the efficient analysis of complex datasets (16). The integration of electronic medical records (EMRs) has facilitated the development of extensive patient databases, including continuous clinical variables, which accelerates the application of AI in medical research and practice. Consequently, predictive models leveraging AI have been developed to enhance clinical decision-making (17). Among the most informative predictors are continuous cardiorespiratory variables, which are routinely monitored in intensive care settings (18). Fluctuations in these vital signs have been shown to reflect the severity of clinical insults (19).

This study aims to utilize digital biomarkers derived from bedside physiologic data collected during the 72-h period of therapeutic hypothermia (TH) to develop a predictive model for identifying neonates with brain injury. Specifically, we seek to stratify neonates into two categories—none to mild or moderate to severe brain injury—based on MRI findings. Continuous bedside clinical data, including heart rate, blood pressure, cerebral oxygen saturation, and systemic oxygen saturation, were extracted from the electronic medical records. We hypothesize that AI-based learning algorithms will accurately classify neonates into the appropriate injury group based on these parameters. The ultimate goal is to develop a model that can detect hypoxic-ischemic brain injury in real time using bedside clinical data, equipping clinicians with objective information to enhance decision-making during the 72-h TH period.

## 2 Methods

### 2.1 Patient populations

#### 2.1.1 NE subjects

This study was approved by the University of Florida Institutional Review Board, and informed consent was obtained from the parents of all neonates eligible for TH within 72 h of birth for inclusion in the Florida Neonatal Neurologic Network registry, as previously reported (3, 20). Eligibility criteria for the hypothermia protocol included a gestational age of 35 weeks or greater, birth weight of at least 1.8 kg, and initiation of therapy within 6 h of birth. Enrolled neonates exhibited signs of encephalopathy, defined by either seizures or abnormalities in three out of six categories on the modified Sarnat exam: level of consciousness, spontaneous activity, posture, tone, primitive reflexes (suck and Moro reflexes), and autonomic system findings (pupil reactivity, heart rate, and respirations). Evidence of hypoxic–ischemic injury was determined by one of the following: (1) a pH ≤ 7.0 and/or base deficit <16 mmol/L, (2) a pH between 7.01 and 7.15 and/or base deficit between 10 and 15.9 mmol/L, or (3) the absence of blood gas results but a report of an acute perinatal event, such as cord prolapse, heart rate decelerations, or uterine rupture. Therapeutic hypothermia was administered using the CritiCool™ blanket device (Mennen Medical Corp., Feasterville-Trevose, PA). Neonates were excluded from the analysis if cerebral oximetry was not performed or if the data were unavailable for review.

### 2.2 Integrated data repository retrieval

To facilitate comprehensive data collection and analysis for all enrolled infants, we utilized the Integrated Data Repository (IDR) in collaboration with the University of Florida Clinical and Translational Science Institute (UF CTSI). The IDR aggregates clinical data from the electronic health record (EHR), incorporating both structured data (e.g., demographics, clinical variables, laboratory results) and unstructured data (e.g., clinical notes, bedside physiologic monitoring). All retrieved data underwent systematic cleaning and preprocessing using R Studio to ensure data integrity and consistency prior to analysis. This thorough data management process allowed the integration of the physiologic variables into machine learning models, creating a complete and accurate physiologic profile for each infant.

In addition to continuous monitoring with the cardiorespiratory physiologic monitor, regional cerebral oxygenation was measured using INVOS™ (Medtronic, formerly Covidien, Minneapolis, MN) Cerebral/Somatic Oximetry Infant-Neonatal Sensors. These sensors utilize near-infrared spectroscopy (NIRS) to monitor regional mixed venous-arterial saturations. Sensors were placed on either the left or right side of the neonate's forehead, and data were continuously collected throughout the hypothermia phase, during the rewarming period, and immediately post-rewarming. This comprehensive monitoring approach provided a detailed dataset for analysis, capturing both systemic and cerebral physiologic parameters.

### 2.3 MRI scoring

MRI were performed at either 3–5 (*n* = 18) days of age following rewarming or 7–12 days of age (*n* = 7, not stable for imaging at 3–5 days of age). One subject had an MRI performed at day 1 of life and another subject at day 20. Neonates were imaged on a Siemens Magnetom Verio 3 T scanner (Siemens, Malvern, PA) at UF Health Gainesville. A single blinded subspecialty board-certified neuroradiologists with over 10 years of experience in neonatal imaging interpreted all the MRI images using the Weeke scoring system (21). The Weeke scoring system evaluates brain injury across three regions: deep grey matter, white matter/cortex, and the cerebellum, with an additional subscore assessing the presence of intraventricular hemorrhage (IVH), subdural hemorrhage (SDH), and cerebral sinovenous thrombosis (CSVT). Each anatomical region is systematically scored based on the extent and distribution of injury. The deep grey matter subscore, with a maximum of 23 points, assesses the thalamus, basal ganglia, posterior limb of the internal capsule (PLIC), brainstem, perirolandic cortex, and hippocampus. Injury in these areas is scored as 0 (no injury), 1 (focal injury affecting <50%), or 2 (extensive injury affecting ≥50%). Additionally, injuries are noted as either unilateral (score of 1) or bilateral (score of 2). The white matter/cortex subscore, with a maximum of 21 points, evaluates damage to the cerebral cortex, cerebral white matter, optic radiations, corpus callosum, punctate white matter lesions (PWML), and parenchymal hemorrhage. Injury is scored as 0 (no injury), 1 (focal injury involving one lobe), or 2 (extensive injury affecting multiple lobes). The cerebellum subscore, with a maximum of 8 points, examines lesions or hemorrhages within the cerebellum. A score of 0 is given for no injury, 1 for focal lesions smaller than 0.5 cm, and 2 for extensive lesions larger than 0.5 cm or multiple lesions. In addition to these anatomical regions, the Weeke score incorporates an “Additional” subscore assessing the presence of IVH, SDH, and CSVT. For each of these conditions, the scoring is binary: 0 for absence and 1 for presence, contributing to a maximum additional score of 3. The total score is calculated by summing the scores from the grey matter, white matter/cortex, cerebellum, and additional categories, with a maximum score of 55. If 1H-MRS data are available, abnormalities in the basal ganglia and thalamus, such as reduced N-acetyl aspartate (NAA) or elevated lactate peaks, are incorporated into the grey matter subscore, increasing the total score to a maximum of 57.

### 2.4 Data pre-processing

The dataset consisted of physiological biomarkers from 138 subjects monitored over 72 h during therapeutic hypothermia. The recorded biomarkers included heart rate (HR), systemic oxygen saturation (SpO₂), cerebral oximetry (rcSO2), arterial systolic blood pressure (ArtSBP), arterial diastolic blood pressure (ArtDBP), and mean arterial pressure (MAP). Some measurements were missing throughout the data collection process. To address these gaps, missing values were imputed using the average of the five nearest neighboring timestamps. Each biomarker feature was standardized using *Z*-score normalization, transforming the data into a standard normal distribution with a mean of zero and a standard deviation of one.

Our dataset had small, randomly distributed gaps due to the retrospective nature of this study and the inclusion of out-born infants, which resulted in occasional missing measurements. Most abnormal physiological events were concentrated early in the monitoring period, whereas the majority of the data reflected stable physiological patterns. We specifically chose the five-nearest-neighbor averaging imputation method due to the following advantages: (1) Local Adaptability: Unlike simpler methods such as forward-fill or global linear interpolation, averaging the five closest temporal neighbors (including timestamps before and after a missing point) preserves local temporal context and prevents oversmoothing; (2) Outlier Resilience: Utilizing multiple neighboring points reduces the likelihood of propagating single-point measurement errors or outliers, a risk inherent in methods like forward-fill; (3) Trend Preservation: Local averaging is capable of accurately capturing subtle, short-term trends and variations in the signal, maintaining physiological realism more effectively than linear interpolation, especially when abrupt physiological changes occur.

We carefully considered alternative methods, including multiple imputation (MI), model-based imputation techniques (such as mixed-effects models), random forest-based approaches, and deep learning methods and evaluated the pros and cons. Multiple Imputation and Model-based Approaches: Methods like multivariate linear mixed-effects models are robust for multilevel or longitudinal data, leveraging hierarchical structures to mitigate bias. However, these methods require assumptions about data distributions and sufficient variability in hierarchical structures, which may not align well with our minute-level physiological data. Random Forest Imputation: Although flexible and capable of capturing nonlinear relationships, random forest-based methods may introduce biases when handling skewed physiological variables or complex interactions inherent to clinical biomarkers. Deep Learning Methods: These models excel with extensive datasets, where substantial training data are available. Given our limited dataset size and small gap intervals, deep learning models are not feasible, as they typically require large training samples and may not reliably capture the short-duration gaps accurately.

After evaluating these methods' strengths and limitations relative to our dataset's characteristics and clinical context, we concluded that the five-nearest-neighbor averaging approach is most suitable for accurately and reliably filling small gaps in our time-sensitive physiological data (22).

### 2.5 AI analysis

We built a Long-Short Term Memory (LSTM) model to predict no-to-mild vs. moderate-to-severe brain injury on MRI, based on the time sequence biomarker features. LSTM is a specialized type of recurrent neural network (RNN) designed to overcome the limitations of classic RNNs, particularly the vanishing gradient problem, by preserving information over long temporal spans through its gated memory cell architecture. In this study, we chose LSTM over the alternative architectures such as Gated Recurrent Unit (GRU) due to its enhanced ability to capture long-range temporal dependencies, which are essential for early diagnosis. Although GRU offers faster training and a simpler structure, LSTM provides finer control over memory retention—critical in our application, where diagnostic accuracy depends on the length of observation (e.g., 24 h vs. 48 h) (23).

There are three major components in the LSTM model: (1) the forget gate ($F_{t}$) which will decide whether we should keep the information from the previous timestamp or forget it. Then, a sigmoid function is applied over it. That will make ft a number between 0 and 1; (2) the input gate ($I_{t}$) which will decide which of the values from the inputs is to be used to change the memory. The sigmoid function determines whether or not to allow 0 or 1 values through. In addition, using the tanh function, you can assign weights to the data, determining their importance on a scale of −1 to 1, and 3) the output gate ($O_{t}$) which will generate final output based on the input and memory of the block. When the sigmoid function is used, it determines whether the 0 or 1 value should be allowed through. Given *h* hidden units, and the batch size is *n*, and the dimension of inputs is *d*. We will introduce the function of each gate in the following:

$$F_{t}=\sigma (X_{t}W_{xf}+H_{t-1}W_{hf}+b_{f})$$

$$I_{t}=\sigma (X_{t}W_{xi}+H_{t-1}W_{hi}+b_{i})$$

$$O_{t}=\sigma (X_{t}W_{xo}+H_{t-1}W_{ho}+b_{o})$$

Where,

$X_{t}\in R^{n\times d}\;$ : input to the current timestamp *t*.

$W_{xf},\;W_{xi},\;W_{xo}\in R^{d\times h}\;$: weight associated with the input.

$H_{t-1}\in R^{n\times h}\;$ : the hidden state of the previous timestamp t−1.

$W_{hf},W_{hi},\;W_{ho}\in R^{h\times h}$ : the weight matrix associated with hidden state.

$b_{f},b_{i},\;b_{o}\in R^{1\times h}$ : the bias.

The LSTM model consists of a single LSTM layer with 64 hidden units for temporal feature extraction, followed by fully connected layers serving as the classifier. Given the limited cohort size relative to the model complexity and training duration, there is an inherent risk of overfitting, where the model may memorize training data instead of learning generalizable patterns. To mitigate this, we employed several overfitting prevention strategies. First, we reduced model complexity by using only one LSTM layer in feature extraction layer. Second, we introduced L2 regularization (weight decay = 1e-2) in the optimizer to penalize large weights. Third, we incorporated early stopping via validation monitoring with patience of 10 epochs. We split the dataset into training, validation, and test sets with a 70%/15%/15% stratified sampling strategy within each class, ensuring balanced representation across sets. Given that the duration of the time-series input plays a critical role in diagnostic performance, we further explored the trade-off between input length and predictive accuracy. Specifically, we compiled two datasets using 24-h and 48-h observation windows, and trained LSTM models on each to compare performance. Models were trained for up to 1,500 epochs using stochastic gradient descent (SGD) with a learning rate of 0.01 and momentum of 0.8. Each setup was trained 10 times and the average accuracy and standard deviation on the test were reported to ensure a robust evaluation. The implementation was developed in PyTorch and executed on a system running Ubuntu 22.04 with an NVIDIA RTX 4080 GPU.

## 3 Results

### 3.1 Patient demographics and study profile

A total of 138 subjects were included in this cohort analysis (39% outborn). Seventy-three neonates (52.9%) exhibited moderate to severe injury on MRI, while 65 neonates (47.1%) had no or mild injury. Both groups were similar in several key characteristics. The majority of neonates were male (60% in the moderate/severe group vs. 54% in the no/mild group). Gestational age was comparable between the groups (38.2 ± 2 weeks for both), as was birth weight (3,212 ± 740 g for the moderate/severe group vs. 3,219 ± 752 g for the no/mild group). Additionally, the incidence of cesarean delivery was similar (56% in the moderate/severe group vs. 65% in the no/mild group), as were umbilical cord gas pH and base deficit values.

Notably, a sentinel event was reported in 43% of the neonates in the no/mild group compared to 33% in the moderate/severe group. One-, five-, and ten-minute Apgar scores did not differ significantly between the groups. However, neonates in the moderate/severe group had a higher incidence of seizures (49% vs. 14%) and a greater proportion with Sarnat scores of III on the initial neurologic examination (37% vs. 8%) compared to those in the no/mild group (*p* < 0.05). For a detailed description of the neonatal characteristics, please refer to Table 1.

**Table 1:** The demographical and clinical characteristics of NE neonates (*n* = 138).

Infant characteristics at enrollment	NE (*n* = 65)	NE (*n* = 73)
	No/mild injury on MRI	Moderate/severe injury on MRI
Sex (%)		
Female	46	40
Male	54	60
Gestational age in weeks (mean ± SD)	38 ± 2	38 ± 2
Birth weight in grams (mean ± SD)	3,219 ± 752	3,212 ± 740
Apgar score at 1 min (mean ± SD)	2 ± 2	2 ± 2
Apgar score at 5 min (mean ± SD)	4 ± 2	4 ± 2
Apgar score at 10 min (mean ± SD)	5 ± 3	5 ± 3
Sentinel event *n* (%)	28 (43%)	24 (33%)
C- section delivery *n* (%)	42 (65%)	41 (56%)
*Respiratory support *n* (%)	55 (85%)	70 (96%)
*Ventilator *n* (%)	44 (68%)	61 (84%)
CPAP *n* (%)	5 (8%)	8 (11%)
*Room Air *n* (%)	10 (15%)	3 (4%)
*Inototropic support *n* (%)	22 (34%)	44 (60%)
*History of seizures *n* (%)	9 (14%)	36 (49%)
SARNAT score II *n* (%)	31 (48%)	25 (34%)
*SARNAT score III *n* (%)	5 (8%)	27 (37%)
Umbilical cord arterial pH (mean ± SD)	6.96 ± 0.18	6.96 ± 0.18
Umbilical cord arterial deficit (mean ± SD)	−15 ± 6	−16 ± 6
Initial pH (mean ± SD)	7.11 ± 0.17	7.11 ± 0.17
Initial base deficit (mean ± SD)	−15 ± 6	−16 ± 6
Initial lactate (mean ± SD)	10 ± 5	11 ± 5
Time to MRI (median [IQR])	4 [4–6]	4 [4–6]
Length of stay (median [IQR])	15 [10–27]	15 [9–32]

NE, neonatal encephalopathy; SD, standard deviation; IQR, interquartile range * *p* < 0.05.

Neonates in the moderate/severe group required respiratory support during hypothermia in 96% of cases, including mechanical ventilation in 84% and CPAP in 11%. In comparison, 85% of neonates in the no/mild group required respiratory support, with 68% receiving mechanical ventilation and 8% on CPAP (*p* < 0.05). Similarly, neonates in the moderate/severe group required ionotropic support during hypothermia in 60% of cases, compared to 34% in the no/mild group (*p* < 0.05).

The median time to MRI was 4 days (IQR: 4–6) for the moderate/severe group and 4 days (IQR: 4–6) for the no/mild group. The median length of hospital stay was 15 days for both groups, with an IQR of 10–27 days for the moderate/severe group and 9–32 days for the no/mild group ( Table 1 ).

### 3.2 Machine learning

#### 3.2.1 MRI injury score cutoff for no/mild and moderate/severe injury

**Figure 1:**
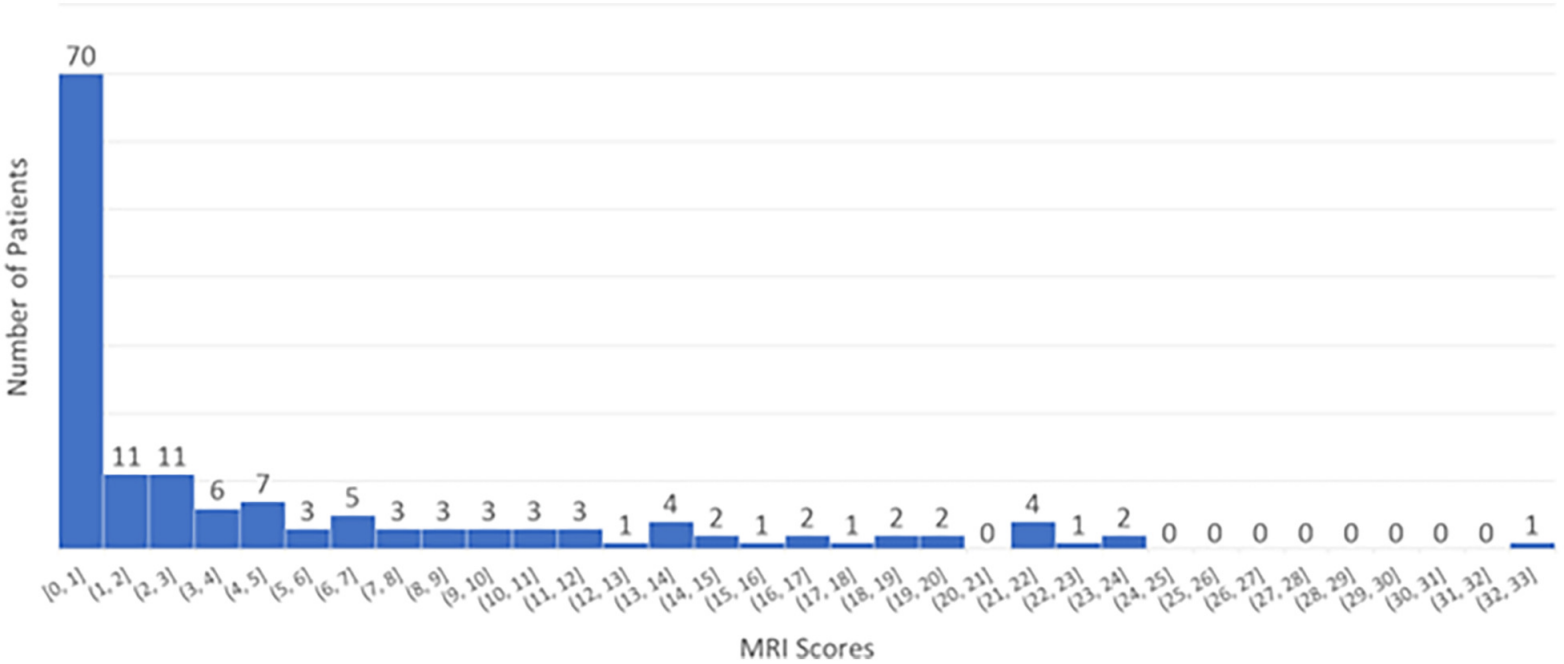
The distribution of MRI scores for all patients.

We analyzed the distribution of MRI scores across all patients, as shown in Figure 1. The majority had an MRI score of 0, while the remaining patients had scores ranging from 1 to 33. Based on these scores, we categorized patients into two groups: (a) no/mild injury (score ≤1) and (b) moderate/severe injury (score >1).

The cutoff of 1 is used to distinguish between normal and abnormal findings: a score of 0 is considered normal, while a score of 1 or higher indicates the presence of abnormality. This threshold is set because even a single point on the Weeke scale reflects a mild detectable injury, which may be clinically significant, where subtle changes are important for prognosis and treatment decisions (24). The high inter-rater reliability (kappa = 0.9) supports the consistency of this scoring method (21). Using this classification, our cohort was approximately evenly divided between normal and abnormal findings.

#### 3.2.2 Analysis of physiologic biomarkers and the impact of data duration on prediction accuracy

**Figure 2:**
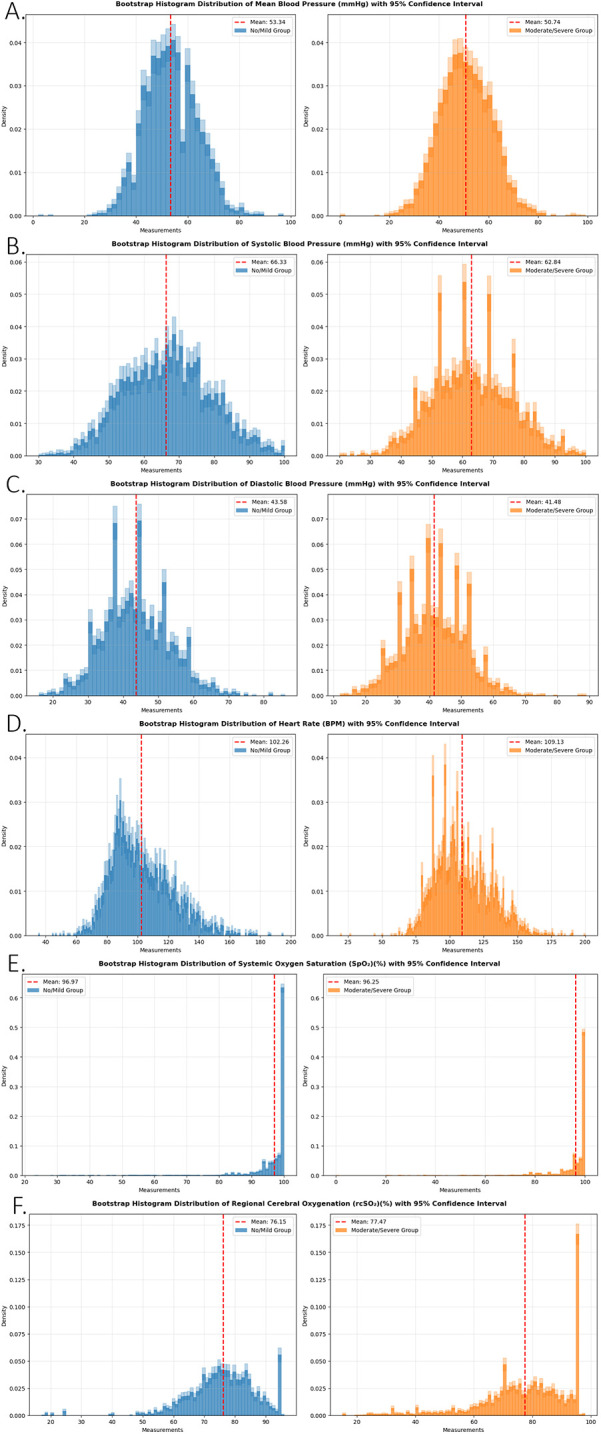
A bootstrap resampling approach was applied to each biomarker within the no/mild injury and moderate/severe injury groups. For each group, data points were resampled 1,000 times to generate bootstrap distributions. Average histograms were computed across the resampled datasets, and 95% confidence intervals were calculated and plotted for each bin. This method enhances visualization of distributional differences between groups and illustrates the uncertainty associated with the estimated frequency of each biomarker. Panels represent **(A)** arterial line mean blood pressure (mmHg), **(B)** arterial line systolic blood pressure (mmHg), **(C)** arterial line diastolic blood pressure (mmHg), **(D)** heart rate (BPM), **(E)** systemic oxygen saturation (SpO₂)(%), and **(F)** regional cerebral oxygenation (rcSO₂)(%).

Six physiologic biomarkers were utilized to train the predictive model: [A] Arterial Line Systolic Blood Pressure (SBP), [B] Arterial Line Diastolic Blood Pressure (DBP), [C] Arterial Line Mean Arterial Pressure (MAP), [D] heart rate, [E] SpO2 and [F] rcSO₂ (cerebral oximetry).Patients were categorized into two groups based on MRI findings: “no/mild” and “moderate/severe” brain injury. To visually assess the distribution of these biomarkers over time, histograms were generated for each group with 95% confidence interval as shown in Figure 2.

Graphical analysis revealed substantial overlap across all measured parameters, with only minor differences between the two groups, except for rcSO₂, which exhibited the clearest distinction between the “no/mild” and “moderate/severe” injury groups. This suggests that cerebral oximetry may serve as a particularly valuable biomarker in differentiating injury severity.

#### 3.2.3 Impact of data collection duration on predictive performance

The duration of physiologic biomarker collection is a critical factor influencing the model's predictive accuracy. A longer monitoring period allows the model to capture more complex temporal relationships and evolving physiological patterns, thereby enhancing its predictive capability. To evaluate the effect of duration, we trained the model using two distinct time frames:

1. First 24 h post-birth—Model trained using all six physiologic biomarkers.
2. First 48 h post-birth—Model trained using all six physiologic biomarkers.

Each model configuration was trained 10 times, with the average accuracy and standard deviation recorded in Table 2. The results demonstrated a significant improvement in predictive accuracy with a longer monitoring period. The 48-h dataset yielded an average test accuracy of 91.2%, compared to 84.6% for the 24-h dataset. This finding highlights the importance of prolonged physiologic data collection in optimizing model performance, reinforcing the need for extended monitoring in neonates at risk for brain injury.

**Table 2:** Comparison of the model performance on test set with duration of 24 h and 48 h.

Duration	Accuracy on test set
24 h	84.6% ± 3.2%
48 h	91.2% ± 3.2%

#### 3.2.4 Evaluation of physiologic biomarkers in predicting MRI-detected brain injury: an ablation study

To assess the relative importance of each physiologic biomarker in predicting MRI-confirmed brain injury, we conducted an ablation study. In this analysis, we systematically removed individual physiologic biomarkers from the predictive model while maintaining a consistent model architecture, parameter settings, and dataset. This approach enabled us to quantify the contribution of each biomarker to the overall predictive performance.

Six different physiologic biomarker combinations were evaluated:

1. Exclusion of Arterial Line Systolic BP—Model trained with Arterial Line Diastolic BP, Arterial Line Mean Arterial Pressure (MAP), heart rate, SpO₂, and rcSO₂ (cerebral oximetry).
2. Exclusion of Arterial Line Diastolic BP—Model trained with Arterial Line Systolic BP, Arterial Line MAP, heart rate, SpO₂, and rcSO₂.
3. Exclusion of Arterial Line MAP—Model trained with Arterial Line Systolic BP, Arterial Line Diastolic BP, Heart rate, SpO₂, and rcSO₂.
4. Exclusion of Heart rate—Model trained with Arterial Line Systolic BP, Arterial Line Diastolic BP, Arterial Line MAP, SpO₂, and rcSO₂.
5. Exclusion of SpO₂—Model trained with Arterial Line Systolic BP, Arterial Line Diastolic BP, Arterial Line MAP, Heart rate, and rcSO₂.
6. Exclusion of rcSO₂—Model trained with Arterial Line Systolic BP, Arterial Line Diastolic BP, Arterial Line MAP, Heart rate, and SpO₂.

Each model configuration was trained 10 times with random initialization to ensure robustness, and the average accuracy along with standard deviation on the test set was recorded (Table 3). The baseline model, which incorporated all biomarkers over the first 48 h of data, served as a reference for comparison.

**Table 3:** The average and standard deviation of accuracy on test set for different combinations of biomarkers.

Biomarker	Mean (Std) no/mild group	Mean (Std) moderate/severe group	*p*-value
rSO^2^	76 ± 11	77 ± 15	0.99
SpO^2^	97 ± 8	96 ± 8	0.11
Heart rate	102 ± 20	109 ± 20	0.77
Systolic blood Pressure	66 ± 12	62 ± 13	0.003
Diastolic blood Pressure	43 ± 9	41 ± 10	0.96
Mean blood Pressure	53 ± 10	51 ± 11	0.93

Biomarker comparison table for Figure 2.

Our findings demonstrate a notable decline in predictive accuracy when specific biomarkers were excluded, reinforcing their importance in model performance. Notably, the absence of Heart rate resulted in the most pronounced reduction in accuracy, identifying it as the most critical biomarker in predicting MRI-detected brain injury. The remaining biomarkers-Arterial Line Systolic BP, Arterial Line Diastolic BP, SpO₂, and rcSO₂-demonstrated comparable effects on predictive performance, suggesting a similar level of importance in the model's decision-making process. These results underscore the varying degrees of influence that individual biomarkers exert on model performance and highlight the necessity of multimodal physiologic monitoring for optimizing early detection of brain injury in neonates.

**Figure 3:**
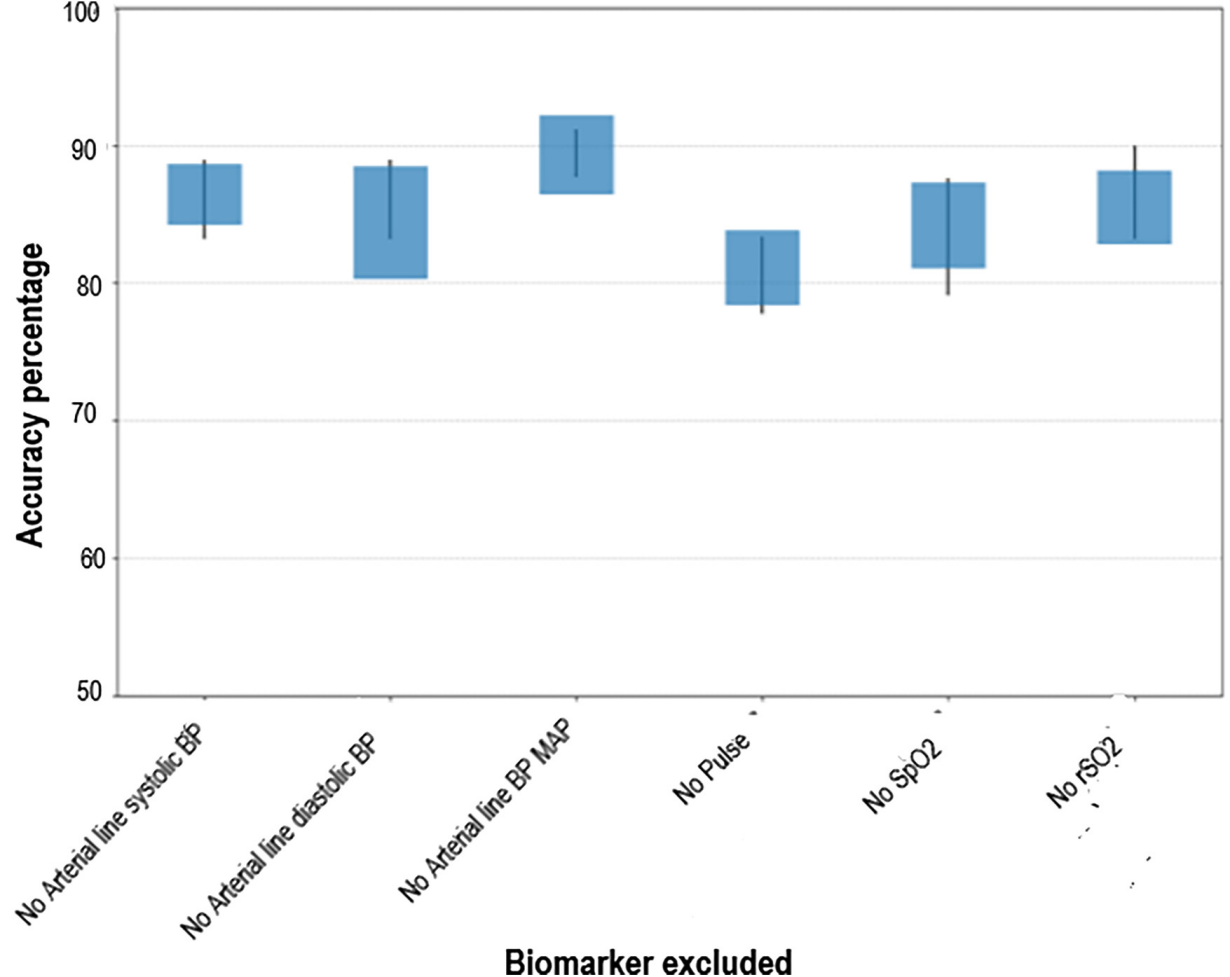
Candlestick graph illustrating the variability and range across multiple experiments. The vertical wicks represent the minimum and maximum observed values, while the bodies show the mean ± standard deviation, offering insights into the consistency of results.

To further evaluate the contribution of individual biomarkers to model performance, a candlestick graph was generated (Figure 3). The *x*-axis represents the biomarker excluded from the training dataset. The candle bodies illustrate the mean ± standard deviation of prediction accuracy across multiple training iterations, reflecting the consistency of the model's performance. The vertical wicks indicate the range between minimum and maximum prediction accuracies, with the upper limit representing the best potential performance for each feature set. The baseline model, trained with all biomarkers from the first 48 h of data, served as the reference for comparison. Our analysis revealed a decline in prediction accuracy when specific biomarkers were excluded, underscoring their importance in model performance. For instance, the absence of heart rate resulted in the most substantial accuracy drop, identifying it as the most critical biomarker. Conversely, excluding Arterial Line BP MAP had minimal impact, suggesting it is the least critical biomarker. Additionally, Arterial Line Systolic BP and Arterial Line Diastolic BP appeared to provide overlapping information with Arterial Line BP MAP. The remaining biomarkers—Arterial Line Systolic BP, Arterial Line Diastolic BP, SpO^2^, and rcSO^2^—demonstrated relatively similar effects on the model's performance, indicating comparable importance. Overall, this analysis highlights the varying contributions of individual biomarkers to the model's predictive capability.

## 4 Discussion

In this study, we evaluated six physiological biomarkers and their predictive capability for MRI-detected brain injury using machine learning. Our findings demonstrate that machine learning models can accurately classify MRI injury status at both 24 and 48 h of age based on physiological data, underscoring their potential for early identification of neonatal brain injury. The incorporation of physiological biomarkers offers an objective, quantifiable approach that minimizes interobserver variability and potential bias associated with traditional clinical assessments such as the Sarnat examination and Apgar scores.

Machine learning is increasingly being applied to neonates with Neonatal encephalopathy (NE)) to develop predictive models by analyzing clinical variables. Prior studies have leveraged machine learning to assess the ability of clinical factors to predict neurodevelopmental outcomes, as measured by the Bayley Scales of Infant Development at one year of age or later (25). In these models, key clinical predictors identified at the time of admission included HIE severity, epinephrine administration in the delivery room, respiratory support, and an initial fraction of inspired oxygen (FiO₂) of 0.21. Additional variables associated with neurodevelopmental outcomes during the hospital course included the severity of EEG abnormalities, the use of steroids for blood pressure management, and the presence of significant brain injury on MRI (25). Machine learning, particularly the random forest algorithm, has also been utilized to improve NE risk prediction by analyzing 72 h of routinely collected clinical data (13). This approach streamlined decision-making for therapeutic interventions by reducing the number of clinical predictors from 154 to 10–12 key variables (13). Our study differs from previously published reports in that it did not incorporate clinical variables but instead focused solely on bedside physiological data as predictive biomarkers. This distinction highlights the potential for real-time, unbiased physiologic monitoring to inform early prognostication in neonates with NE.

Our ablation study identified heart rate as a key physiological variable in our predictive model. Several studies, including our own, have demonstrated the ability of heart rate variability (HRV) to predict MRI-detected brain injury and neurodevelopmental outcomes (26–29). Although speculative, our findings may reflect alterations in sympathetic-parasympathetic tone following hypoxic-ischemic (HI) injury, potentially influencing resting heart rate. However, further research is needed to validate this proposed mechanism. Additionally, the higher use of inotropes in the moderate/severe group may have also impacted heart rate. Initial visual data suggested that cerebral regional oxygen saturation (rcSO₂) might serve as an important discriminative variable, a hypothesis supported by early iterations of our model. However, our ablation study did not confirm its significance in the final model. Interestingly, previous studies examining regional saturation trends have shown that machine learning models incorporating these trends were associated with MRI outcomes, whereas models utilizing mean absolute values of rcSO₂ were not (30). We hypothesize that the reason our ablation study did not confirm the significance of rcSO2 in the final model is due to rcSO2 often being influenced by systemic factors(carbon dioxide levels, hypotension, pressors, and sedation), timing of injury in infants with no sentinel event recorded, as well as the effects of therapeutic hypothermia on cerebral metabolism and blood flow, which exhibit high inter-individual variability (31).

Several potential limitations exist in our study. First, the data were retrospective and reliant on documentation in the medical record, which may introduce variability and potential inaccuracies. Additionally, data granularity varied among subjects, with higher-acuity patients having more frequent and prolonged vital sign recordings compared to lower-acuity patients. Missing data were also a challenge, particularly for out born neonates, as physiologic data collection did not begin until the time of admission to UF Health. To address these limitations, future studies will incorporate a real-time data capture device capable of recording bedside physiological data every 30 s. This approach will enhance data consistency and resolution, reducing reliance on manually recorded values. Furthermore, implementing this portable device during neonatal transport will allow for continuous physiologic monitoring, mitigating data loss in transferred patients and improving the accuracy of predictive modeling. Finally, the generalizability of the model to other institutions with different patient populations, monitoring equipment, or clinical practices would require further validation in multicenter studies.

Our study, along with existing literature, highlights the potential of machine learning (ML) in leveraging physiologic variables, EEG findings, and clinical parameters to enhance the management of hypoxic-ischemic encephalopathy (HIE). By providing clinicians with data-driven, actionable insights, ML-based models facilitate earlier intervention, individualized risk stratification, and optimization of neuroprotective strategies. The integration of ML into neonatal care has the potential to transform clinical decision-making, ultimately improving outcomes for neonates with HIE.

## Data Availability

The original contributions presented in the study are included in the article/Supplementary Material, further inquiries can be directed to the corresponding author.
